# Annotated textual dataset PV600 of perovskite bandgaps for information extraction from literature

**DOI:** 10.1038/s41597-025-05637-x

**Published:** 2025-08-11

**Authors:** Matilda Sipilä, Farrokh Mehryary, Sampo Pyysalo, Filip Ginter, Milica Todorović

**Affiliations:** 1 University of Turku, Department of Mechanical and Materials Engineering, Turku, 20014 Finland; 2 University of Turku, TurkuNLP, Department of Computing, Turku, 20014 Finland

**Keywords:** Computational methods, Solar cells

## Abstract

Scientific literature provides a variety of experimental and theoretical data which, if extracted, could offer new opportunities for data-driven discovery in materials research. Natural language processing (NLP) tools enable information extraction (IE) of structured information from unstructured text. The performance of IE tools needs to be systematically evaluated on manually annotated test datasets, but there are few publicly available annotated materials science datasets and none on perovskites, promising materials for photovoltaics. We present a perovskite literature dataset with 600 text segments extracted from an open access manuscript corpus. The PV600 dataset focuses on five inorganic and hybrid perovskites and contains 227 manually annotated bandgap values identified from 188 segments. Moreover, we recorded the bandgap type, whether it was experimental, computational, from the literature, or from unknown source. To demonstrate the intended use of the dataset, we applied it to evaluate the IE performance of a question answering (QA) method, a rule-based method, and generative language models (LLMs). We exhibit a further application in testing segment preselection with LLMs in IE.

## Background & Summary

The field of materials science has witnessed a rapid expansion in the number of scientific publications, generating an increasingly large amount of valuable textual data. By extracting and analysing information from text with NLP tools, researchers can identify research trends^1^, classify materials synthesis methods^2^ and even predict new thermoelectric materials^3^. The key NLP task with the materials science text is information extraction (IE), where the aim is to extract structured information from the unstructured text.

During the past decade, IE approaches have undergone significant development. Early methods feature predefined grammatical rules to recognise sentence structures and extract information, and are encoded in tools like LeadMine^4^, ChemicalTagger^5^ and the state-of-the-art method ChemDataExtractor2 (CDE2)^6^. Supervised machine learning (ML) techniques are also used, which depend on manually annotated training datasets to train models for identifying specific entities or relationships in text: a labor-intensive process that leads to highly accurate models. For example, supervised ML models were trained to extract the synthesis procedures of materials^7^ and the phase-property relationships of aluminium alloys^8^. More recently, question-answering (QA) models have shown promising results in materials science IE^9,10^ by exploiting transfer learning from BERT architectures rather than single-shot training. Lastly, the rapid evolution of LLMs has also facilitated their IE application on topics such as solid-state impurity dopings^11^, metal-organic framework properties^11–13^, and bulk moduli^14^. LLMs were also used to predict the synthesisability of inorganic compounds^15^, to collect a large dataset of reticular chemistry questions and answers^16^ and to develop AI-agents for extracting various materials science information^17^.

Ensuring the reliability of the text-extracted information is critical for decision making in materials design. IE methods have diverse challenges, such as the lack of domain-specific knowledge, difficulty in addressing rarely mentioned information, and potential bias in output, originating from the bias in the training dataset of the method. Evaluating the performance of IE tools requires a human-annotated gold standard dataset that serves as a benchmark. In manual annotation, one or more domain experts analyse a predefined set of texts and mark entities of interest, and potentially their relationships to each other. To systematically evaluate IE tools in materials science, diverse annotated benchmark datasets are essential, because the field is broad, and many research areas have their own unique terminology and concepts. To date, annotated datasets were generated for paragraphs of synthesis procedures^18^, solid oxide fuel cell full text articles^19^ and inorganic materials science full text articles^20^. In this study, we provide a dataset that addresses numerical property values of perovskites, technologically-relevant materials under intense research, and with no benchmark datasets to date.

**Fig. 1:**
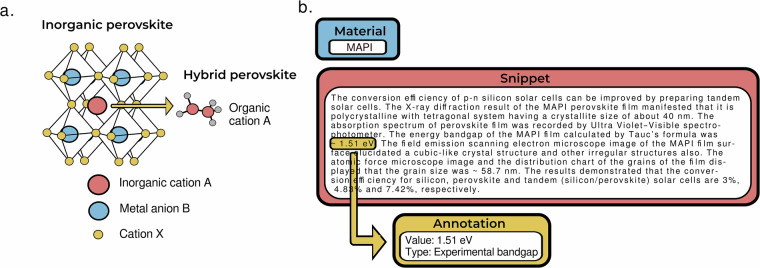
Perovskite unit cell and an annotated snippet. (**a**) An example unit cell of inorganic perovskite and hybrid perovskite. (**b**) An example snippet extracted for MPI material, annotated bandgap value and the bandgap type. In the snippet (from the article^58^) the material name MAPbI_3_ has been standardised to MAPI.

Perovskites are chemically diverse crystalline materials characterised by the chemical formula ABX_3_, where A and X are cations and anions and B a metal atom. This versatile structure allows for tuning of functional properties through element substitution. Figure 1a illustrates how replacing atoms with small organic molecules considerably broadens the range of functional properties of perovskites, resulting in organic-inorganic (hybrid) perovskites. Perovskites are promising materials for optoelectronics^21,22^, sensors^23,24^ and photodetectors^25,26^. In addition, perovskites are increasingly deployed in photovoltaics because they exhibit high power conversion efficiencies^27–29^, paving the way to more efficient solar cells^30–32^. The community interest into perovskites is highlighted by number of available databases, ranging from manually collected data of over 42,400 perovskite photovoltaic devices^33^ to a database of 515 perovskite compound properties^34^. There is continuing demand for accurate, high quality perovskite datasets^35^. Perovskite bandgap is an important materials property for photovoltaic efficacy: it determines the wavelength range of light absorption and efficiency of electricity generation^36,37^.

In this article, we introduce a manually annotated benchmark dataset PV600 with a focus on bandgap values of perovskite materials, intended for evaluating the performance of IE tools. The dataset was extracted from open access articles in form of text segments referred as snippets, to continue the standard of using text paragraphs as documents in IE datasets. However, unlike existing datasets, we do not focus on author-defined paragraphs but instead search for relevant information and extract the surrounding snippets. Text snippets serve to narrow the focus from full scientific texts towards relevant information, but still maintain more context than single-sentence entries. We manually annotated 600 snippets for bandgap values and their types, to classify whether the values were obtained experimentally, computationally, from the literature, or from an unknown source. Figure 1b displays an example snippet extracted for a particular perovskite, along with the annotated bandgap value and type. We describe the annotation procedure in detail to encourage other colleagues towards annotating further benchmark datasets. The dataset focus is on five well-known inorganic and hybrid perovskite materials in order to achieve statistically significant IE metrics in performance studies. We demonstrate the use of our dataset to evaluate the capabilities of contemporary IE approaches: a QA method, a rule-based method and four generative LLMs. In this way, we aim to promote further comparative studies into the performance of novel language tools in IE tasks for materials science research.

## Methods

To create an annotated dataset, we first extracted and processed the snippets from open access texts. Next, selected snippets were prepared for the annotation procedure, which was carried out by experts following specific guideline. Annotated snippets were processed and organised into the PV600 dataset. We devised a series of IE tests to illustrate dataset application, and below we present selected IE methods, test design and relevant quality metrics.

### Generating the snippet set

The first step in the dataset creation was to acquire a large scale corpus of scientific articles. We downloaded full text manuscripts with the query word ‘perovskite’ from five different journal publishers: Elsevier^38^, Springer^39^, Royal Society of Chemistry^40^, arXiV^41^ and Core API^42^. A total of 238,431 articles were collected between October 2022 and January 2023. After article processing steps like conversion to text format and duplicates removal (following previous work^9^), we obtained 194,322 unique publications. To ensure that the evaluation dataset could be made publicly available, annotations were selected exclusively from open access publications following the procedure described in Supplementary Information (SI) section S1, which resulted in 47,688 full text manuscripts.

We constructed the dataset from snippets because, unlike single sentences, they do not only provide more context but also enable us to identify cross-sentence relationships between the material, property, unit, and value. They are also more efficient to process than full text articles, where most of the text bears no relevance to the IE task. Previous tests^9^ indicated that the 7-sentence snippets around the information of interest (material and property) are long enough to contain all of the necessary information, while limiting unnecessary text^9^. The material and property names can be written in multiple ways, so we standardised them to one representation (e.g. band gap and bandedge  → bandgap). This serves several purposes: it facilitates the annotation task for the annotators, prevents potential misinterpretation of synonyms, and facilitates the comparison between IE methods by reducing task complexity. Snippets were saved as separate files with unique names to facilitate annotation. Each snippet was accompanied by metadata with various information on the source article.

When deciding on the dataset size, we considered two aspects: on the one hand the dataset must be comprehensive enough for IE evaluation, but on the other, annotation is laborious and requires manual work. In previous materials science studies, the size of annotated datasets varied with their intended use. For instance, the annotated materials synthesis dataset contains 230 synthesis procedures, typically formatted as single paragraphs^18^. The SuperMat dataset includes 142 full text publications focused on superconductor material names and critical temperatures^43^ and the solid-state-synthesis dataset of 834 paragraphs has been used to train ML model to extract synthesis parameters^44^. Given that recognising numerical values for materials properties is an easier task than extracting experimental protocols, we proceeded with 600 snippets.

We stratified snippets selection to distribute content equally between five different perovskite materials, resulting in 120 snippets per compound. We concentrated on five different perovskite materials in the annotation dataset: MAPI, FAPI, MAPB, CsPbI_3_ and CsPbBr_3_ (representing both organic and hybrid compounds). Controlling the number of unique materials in the dataset allows for sufficient number of occurrences for each material to draw meaningful statistics and comparisons between materials. We also balanced snippet selection per publisher to avoid any biases that may be associated with different text conversion tools used. After these considerations, 600 snippets were selected at random from the pool of open access publications as detailed in SI (Table S2).

### Annotation procedure

**Fig. 2:**
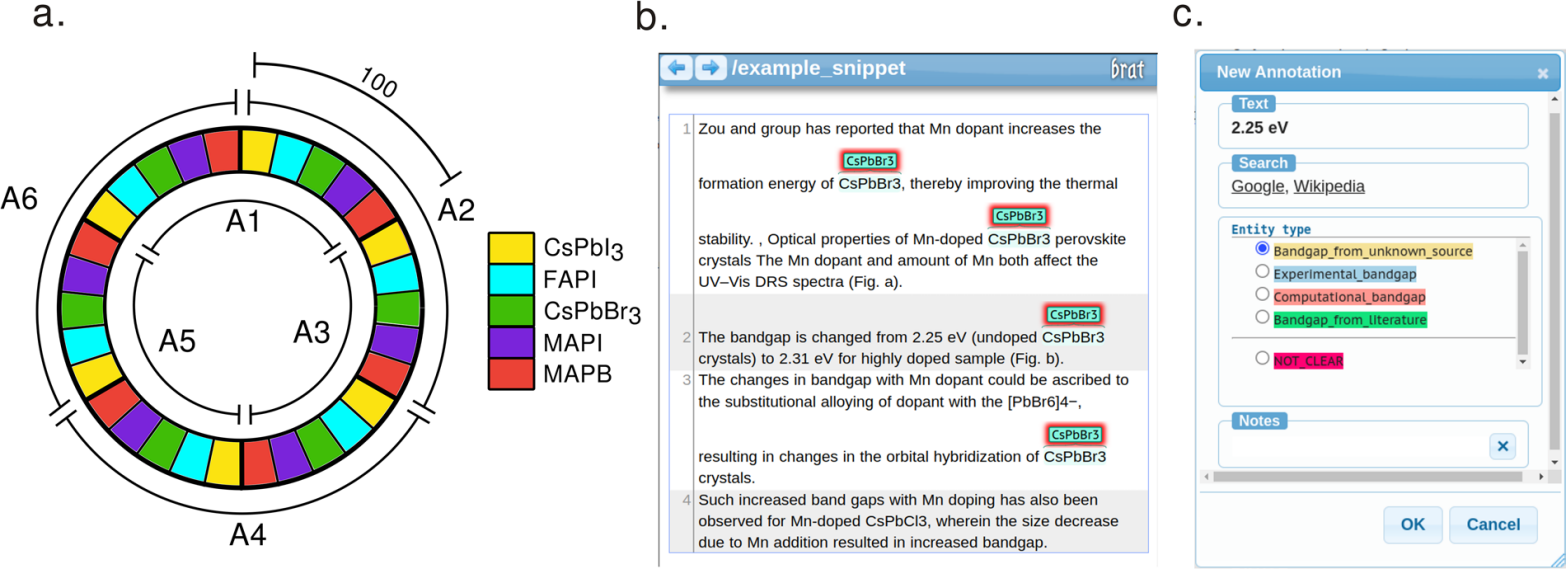
The annotation dataset distribution and *brat* windows. (**a**) The perovskite literature dataset annotations. The colored blocks depict the 40-snippet subgroups of different materials: yellow for CsPbI_3_, blue for FAPI, green for CsPbBr_3_, violet for MAPI and red for MAPB. Annotators are marked as A1 for annotator 1, A2 for annotator 2 etc. Every snippet was annotated by two annotators. (**b**) Labeled snippet (from article^59^) in *brat* annotation tool. Name CsPbBr_3_ is labeled and in this snippet the material name is mentioned four times. (**c**) The annotation dialog of *brat*. From the dialog the user can select bandgap type from four different choices. Under the entity type dialog there is a type of NOT CLEAR answer, which could be selected if the annotator was not sure what to annotate.

We used the open source *brat rapid annotation tool* (*brat*)^45^ for annotation due to its browser-based accessibility and versatility. The tool was hosted on a local server, where all snippets were uploaded and brought accessible to all annotators. We secured a total of six materials science domain experts for this task: a professor, two postdoctoral researchers, two PhD researchers and one MSc student. To improve quality we arranged double annotation, so that each snippet was read through and marked by two annotators. The snippets were distributed randomly into six folders, as presented schematically in Fig. 2a. Each of the six annotators (A1, A2, A3 etc.) had access to 200 snippets (divided into blocks for 5 different materials), where 100 snippets were shared with one annotator and the other 100 with another.

The annotators were provided with instructions and a guideline, which we describe in the SI section S2 and distribute alongside the annotated dataset^46^. Because the dataset is intended for testing the capability IE tools to extract bandgap values, the annotators were to answer the question: *What is the numerical value of bandgap of the material of interest?*. Snippet generation was material-specific, and the material name was highlighted in the *brat* window (Fig. 2b) to facilitate annotation. Experts were asked to select the span of text (answer) that contained the numerical value, or the numerical value and the unit eV if the unit is in the same text span as the numerical value. The eV is by far the most common bandgap unit used today, particularly in recent open access articles. To maintain consistency, annotators were instructed to ignore bandgap values reported in other units.

After selecting the answer, the experts were also asked to identify the type of the value from context and indicate whether the bandgap was an i) experimental bandgap, ii) computational bandgap, iii) bandgap from literature or iv) bandgap from unknown source. To accomplish this, we devised the custom bandgap type menu displayed in Fig. 2c. The experimental and computational values were defined as bandgaps experimentally measured or computed (respectively) in the host article of the snippet.

Bandgaps calculated, measured or otherwise presented in other articles were assigned the literature type. This could be determined by citation numbers, citation parentheses or clear references to other manuscripts. If the bandgap value type could not be concluded given snippet context, the annotators were instructed to select unknown as the bandgap type. In difficult cases, the NOT CLEAR option was provided (Fig. 2c) to mark the snippet for deeper examination and avoid uncertain annotations.

Once the initial annotation round was completed, we performed data analysis to establish good quality final annotations for each snippet. First, the answers from doubly-annotated snippets were checked for any disagreements. An annotation match was established where answers were completely or partially overlapping. In the case of partial overlaps, the final annotation was selected to be the one with a longer text span to minimise possible data loss. The same check was performed on reported bandgap types. Next, the few cases of disagreements and those labelled NOT CLEAR (16 in total) were resolved in meetings between annotators and the annotation lead.

In the last step, we processed the most difficult answers where two values were selected in the same text span. These values belonged either to range (‘1.5 eV to 1.6 eV’ or ‘1.4-1.5 eV’) or were connected with the word ‘and’ (‘1.5 eV and 1.6 eV’). Text spans denoting range were processed into one entity by computing the average value, which is most representative of the range (e.g. ‘1.5-1.6 eV’  → ‘1.55 eV’). Bandgap values connected by the word ‘and’ were introduced as two separate entities. In one case the annotated value consisted from the bandgap value and the error margin, which we processed by removing the error margin.

### Methods for extracting information from text

To demonstrate the intended application of the annotated perovskite literature dataset, we compared the performance of three different IE approaches. The selected methods were the rule-based CDE2^6^, QA method for materials science (QA-MatSciBERT)^9^ and the IE abilities of four LLMs. We selected four free LLMs Mixtral-8 × 7B-v0.1 (Mixtral)^47^, Mixtral-8 × 7B-Instruct-v0.1 (Mixtral-Instruct)^48^, Llama-3.1-8B (Llama-3.1)^49^, Llama3-ChatQA-1.5-8B (Llama3-ChatQA)^50^, and the paid GPT-4o^51^. The QA-MatSciBERT and CDE2 were specifically designed to extract information from the materials science literature, but all LLMs are general, domain-agnostic language models. Further information about the foundations, training and refinement of the models above can be found in SI section S3.

### Test design for comparing the performance of IE methods

Typically, IE tools would be applied to the entire benchmark dataset of snippets to determine their capacity for extracting the bandgaps of materials. However, recent work indicated that previously selecting the snippets that likely contain the information of interest improves the accuracy of the subsequent IE task^12^. Such preselection can be performed with LLMs using a question *Does the following snippet contain the bandgap value of material x?*, where material x is the predefined material of the snippet. The outcome is a snippet classification into useful and empty categories. Only the useful texts which most likely contain the wanted information would be selected for the final IE.

**Fig. 3:**
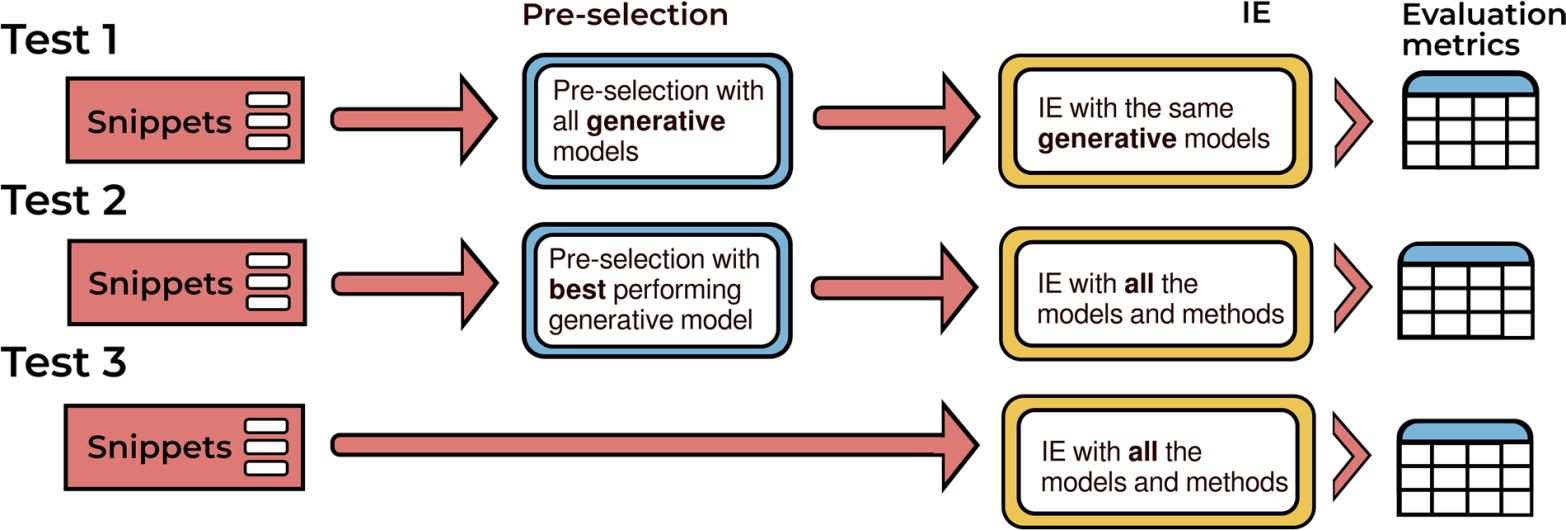
Schematic figure of three IE tests. Test 1 trials snippet preselection followed by IE with the same model, Test 2 features the best-performing preselection tool and Test 3 provides the IE standard test.

To establish whether preselection affects the performance of IE methods and in what implementation, we devised three different tests illustrated in Fig. 3.

In Test 1, we reviewed the performance of five LLMs first on snippet preselection, and next on the subsequent IE. The model that performed the best on the preselection task was deployed to perform the selection in Test 2, after which all IE models were tested on the IE task. Test 3 featured the standard IE protocol, without preselection. For the subset of snippets that contained annotated values, we also tested the best-performing LLM on classifying the bandgap type.

User prompts significantly affect the performance of language models on different tasks. We therefore optimised the prompts, especially because we are using zero-shot prompting. In the zero-shot approach, models are not provided with any task-specific examples before making predictions, simulating their real-world application in IE. The prompts were optimised for the different tasks, adjusted to the needs of the specific study. To determine the optimal prompt for each model, we explored 4 IE prompts with all the LLMs and the QA model, selecting the one that produced the best results for each model (as detailed in SI section S5). A similar approach was followed with the preselection optimisation, but here we optimised the prompt over 8 different options because single words were found to significantly affect the performance of the models. The test with the best-performing LLM to classify the bandgap type was optimised over two prompts with multiple parts.

The evaluation metrics commonly used in IE tasks are precision, recall, and F1-score. Precision measures the proportion of correctly extracted information relative to the total amount extracted by the method. Recall is the proportion of correctly extracted values out of all annotated values. The defining metric F1-score is the harmonic mean of precision and recall. The metrics above required us to define true positive, false positive and false negative outcomes. A true positive was achieved when the extracted value matched the annotated answer numerically (e.g. ‘1.5’ and ‘1.5 eV’). A false positive arose if the extracted value either did not numerically match the annotation or (in the case of a LLM) was hallucinated. A false negative occurred when the IE tool failed to extract the annotated bandgap value and returned no value.

For the subset of snippets that contained annotated values, we also evaluated the capability of the best-performing LLM in classifying the type of bandgap. For this test, we used only snippets containing annotated (gold standard) bandgap values and instructed the language model to determine the type of the annotated bandgap. The evaluation metric for this test was classification accuracy, defined as the proportion of correctly classified types relative to the total classified types.

## Data Records

We present the data records in both the *brat* annotation and csv formats for versatile application. The *brat* annotation format is standard in many NLP datasets and allows easy data integration into IE pipelines. The csv format facilitates data overview and analysis with python or spreadsheets. The files and accompanying metadata were made publicly available^46^.

### Tabular data format

The dataset is organised in a table (*PV600.csv*) with 601 rows, where first row is the header row and each row after it corresponds to one snippet. Table 1 lists the columns categories and descriptions. Each row has 43 columns, where the first 7 entries contain snippet metadata. The following 36 columns are reserved for up to 6 annotated values per snippet, with 6 entries to describe each annotation. If the snippet does not contain 6 annotations the latter columns are empty. In the annotation entries (columns 8-13 in Table 1) the first entry is the actual annotated value. The next two entries are the beginning and ending indices of the annotated value in the snippet, calculated as the number of characters from the beginning of the snippet. After this, (the column 11 in the Table 1) it is defined whether there was a special character (dash, ‘to’,  ± or ‘eV’) in the annotation. If there was, the value was processed to contain only one numerical value (for visualisation purposes) and the processed value was entered in the next column. If the annotated value does not contain any special character, the value in the processed value field is the same as the annotated value. The last entry corresponding to a single annotation is the bandgap type, which could take one of four bandgap type values.

**Table 1:** Description of the columns in the dataset table (*PV600.csv*).

No.	Column name	Description	Example
1	Snippet name	The name of the snippet file	FAPI_10.1016–j.jmrt.2021.03.107_31
2	Article identifier	The DOI or other identifier of the snippet origin article	10.1016/j.jmrt.2021.03.107
3	Publisher	The data provider of the article	Elsevier
4	Year	Publication year of the article	2021
5	Material	Material of the interest	FAPI
6	Text	Full text of the snippet	The strong characteristic diffraction……cells or optoelectronic applications.
7	Annotations	Does the snippet contain annotation (yes or no)	yes
8	Annotation_1	Annotated value number 1	1.5-1.4
9	Start_index_1	Beginning character index of the annotation 1 calculated from the beginning of the snippet	1312
10	End_index_1	Ending character index of the annotation 1 calculated from the beginning of the snippet	1319
11	Special_character_1	Does the annotation contain something else than just pure numbers (e.g. ‘-’)	yes
12	Processed_annotation_1	If annotation denotes range, it is averaged here or the error marking has been removed	1.45
13	Bandgap_type_1	The type of the bandgap	Literature

The columns 8-13 define one annotation in the snippet and the columns after these follow the same structure, where 6 columns correspond to one annotation in the snippet.

### Brat annotation format

With the *brat* annotation tool, it is common to store annotation files separately from the original snippet text files to ensure that the source document remains unaltered. For each snippet (e.g., filename.txt), there is a corresponding annotation file (e.g., filename.ann). The PV600 *brat* folder contains 600 snippets text files in ascii format and their corresponding *.ann files. The filenames are assigned as Material_Identifier_Regex-index.txt. Here, the material denotes the material of interest in the snippet, and identifier the article identifier of the article from which the snippet is selected. The article identifier was most often digital object identifier (DOI) or alternatively arXiV or Core ID. The regular expression index serves to differentiate between snippets from the same article and it was computed as the character position within the snippet where the regular expression fist matched either a material, property, or unit.

Each text file is accompanied by an *.ann file which contains the annotations. There can be from 1 to 6 rows in the *.ann file where each row corresponds to a single annotation in the snippet. Each of these annotation entry row consists of space-separated 5 entries, for example **"T1 Computational_bandgap 506 510 1.70”**. As standard in the *brat* annotation format, the first character T denotes that the row in the .ann file depicts an entity. The number after it (here 1) is the ordinal number of this specific annotation in the snippet and can take values 1-6. Next is the bandgap type, e.g. “Computational_bandgap”. Two numbers, here 506 and 510, are the beginning and ending character indices of the annotated span counting from the beginning of the snippet. The final item in the row is the annotated value itself, which in this case is ‘1.70’. The units were omitted since all annotations were in eV. The *.ann files do not contain any processed values (like in the csv file): the last field lists the annotation verbatim. To facilitate future benchmark comparisons with information extracted from text, it is crucial that the values in the *.ann files remain exactly as they appeared in the original text snippet (potential special characters included).

## Technical Validation

### Dataset statistics

Here, we review the composition and statistics of the annotated bandgap values to better understand the outcomes of IE applied to this dataset.

In total, 227 annotated bandgap values were identified across the 600 segments. They were derived from 188 snippets, where 24 of them contained 2-6 annotated bandgap values. Out of these, examples of snippets with 3, 4, 5 and 6 values occurred only once (rare), but 20 snippets featured 2 values (about 10% of the dataset). All IE tools should be able to extract multiple values from a single snippet. Therefore, including snippets with multiple values is essential for performance testing. Lastly, 412 snippets did not contain any bandgap value, which is important for evaluating IE methods because negative examples help assess false positive predictions.

**Fig. 4:**
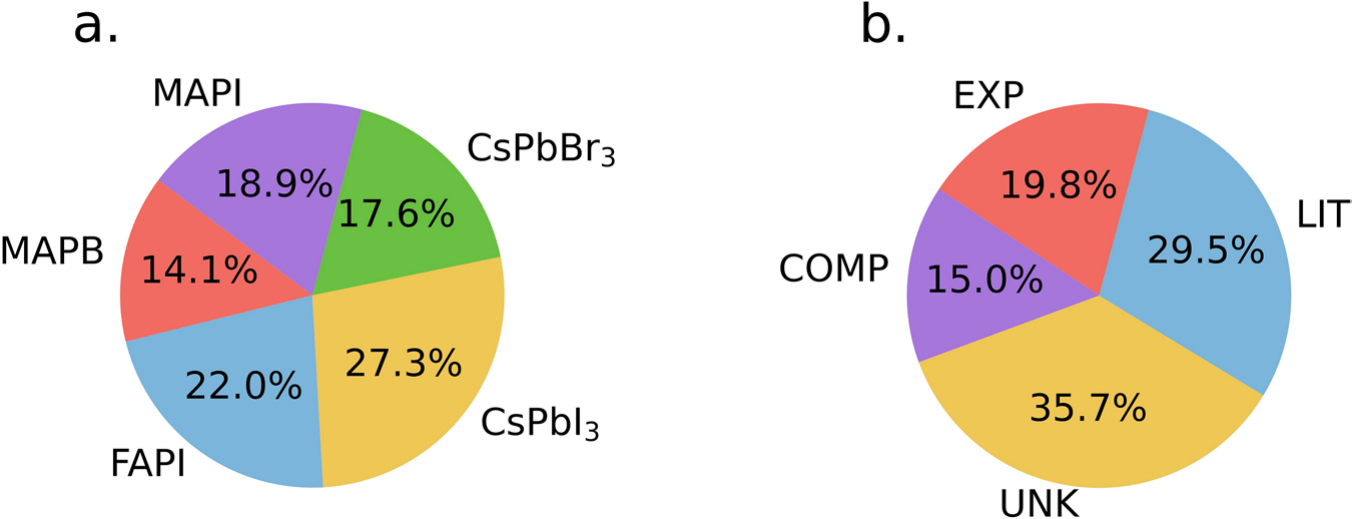
Composition analysis of PV600 dataset. (a) Fractional distribution of annotated values across 5 different materials. (**b**) Fractional distribution of identified bandgap type as follows.

The distribution of annotations classified by material and bandgap type are summarised in Table 2 and illustrated in Fig. 4. The analysis reveals that although we selected equal portions of the dataset to represent different materials, the extracted bandgaps are not equally distributed.

**Table 2:** Number of annotations by material and bandgap type.

Type/Material	MAPI	MAPB	FAPI	CsPbI_3_	CsPbBr_3_	Total
Experimental bandgap (${{\rm{EG}}}_{{\rm{EXP}}}$)	5	12	9	2	17	45
Computational bandgap (EG_COMP_)	5	3	12	13	1	34
Bandgap from literature (EG_LIT_)	11	11	12	22	11	67
Bandgap from unknown source (EG_UNK_)	22	6	17	25	11	81
Total	43	32	50	62	40	227

Most values were annotated for CsPbI_3_ (27.3 %) and least for MAPB (13.1 %) As for bandgap type, 19.8 % of annotated bandgaps were experimental and 15.9 % were computational. Bandgaps of unknown origin were by far the most common at 35.7 %, but values from literature were also well represented with 29.5 %.

**Fig. 5:**
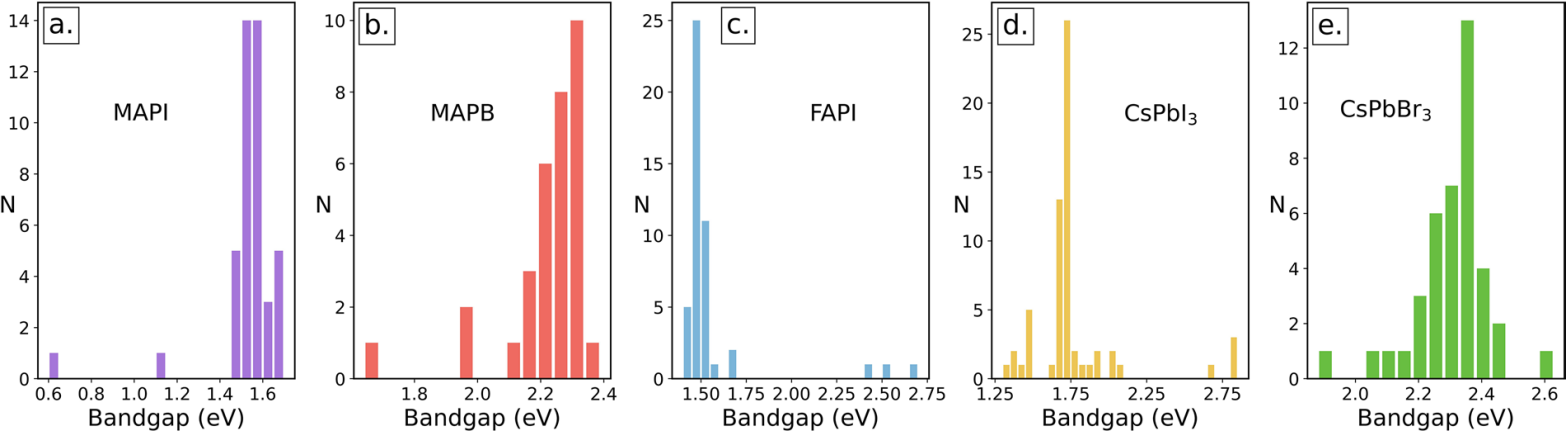
Histograms of gold standard annotated values of material bandgaps EG in electronvolts (eV). The N on the y-axis describes the number of the values. (**a**) MAPI, (**b**) MAPB, (**c**) FAPI, (**d**) CsPbI_3_ and (**e**) CsPbBr_3_.

Typical IE tasks for extracting materials properties result in a statistical distribution of values. These could now be compared with gold standard bangap distributions visualised in Fig. 5 for the 5 different materials. Table 3 presents the statistical description for further comparisons with IE outcomes.

**Table 3:** Histogram statistics of the bandgap distribution extracted from the dataset for the different materials considered in this study.

Material	MAPI	MAPB	FAPI	CsPbI_3_	CsPbBr_3_
Mean	1.54	2.22	1.56	1.77	2.30
Median	1.56	2.24	1.48	1.73	2.32
Mode	1.55	2.30	1.48	1.73	2.30
SD	0.17	0.14	0.27	0.30	0.12
Range	1.10	0.75	1.30	1.52	0.72

The distributions are illustrated in Fig. 5. The SD denotes standard deviation.

All distributions feature a pronounced peak, which reflects either a consensus on bandgap values within the scientific community, a higher frequency of study or both. Additionally, CsPbI_3_ and FAPI demonstrate high peaks, potentially due to more frequent study or recent focus in the literature. High consensus may also stem from inherent material properties or from more contemporary research efforts.

The spread of data varies from the relatively narrow 0.72 eV range for CsPbBr_3_ to the broader 1.52 eV range for CsPbI_3_. This spread reflects variations arising from different measurement techniques, simulation methodologies or material alterations, all of which contribute to uncertainty. To gain insight into the origins of the range, we reviewed several edge cases. The lowest values in the MAPI^52^ and MAPB^53^,^54^ distributions were computational bandgaps (DFT with GGA functionals), but the same could be said of the highest FAPI bandgap^55^ (computed from a cluster model). The highest CsPbI_3_ bandgap was measured in the orthorombic phase, which differs from the bandgap in cubic phase^56^. These findings confirm that annotated values are valid and reliable, but dataset users should expect a similar spread of values in their IE studies.

**Fig. 6:**
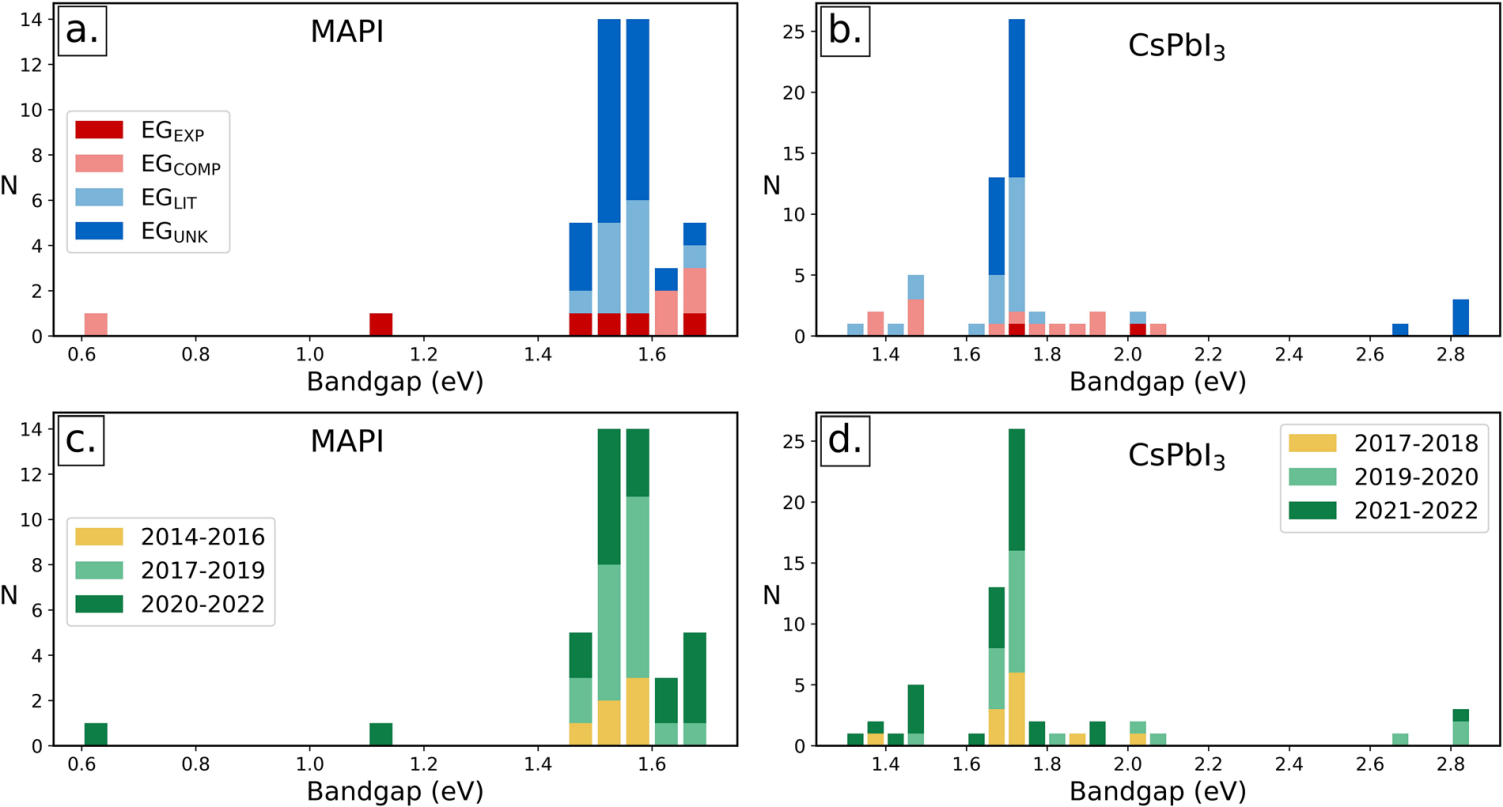
Stacked histograms distributed by type and year. (**a**) Bandgap types of MAPI annotated values. (**b**) Bandgap types of CsPbI_3_ annotated values. (**c**) Yearly distribution of MAPI annotated values. (**d**) Yearly distribution of CsPbI_3_ annotated values.

PV600 dataset additionally enables analyses data classification by bandgap type and year. We demonstrate these possibilities on one hybrid (MAPI) and one inorganic (CsPbI_3_) perovskite in Fig. 6. Bandgap type analysis for both materials (Fig. 6a,b) reveals that experimental values exhibit greater mutual agreement compared to the computational ones. This is expected given that simulation choices critically affect computed values. While DFT simulations are known to underestimate bandgaps, computational bandgaps can be often found on the higher end of the distribution, contrary to our expectations. Literature values are clustered like experimental ones, and this might be because previous experimental measurements are most often cited.

Figure 6c,d reveal the historical investigation of annotated bandgap values in 2-year time intervals. The number of bandgap values increases with time, reflecting both the rising interest in perovskites in recent years and the growing prevalence of open access publishing (strongly represented in this open access dataset). In addition, more recent studies were associated with a greater variability of recorded bandgap values. This contrasts with expectations that older studies would produce a large spread due to less sophisticated methodologies. We observed the same bandgap type and historical measurement trends for the hybrid and inorganic perovskite materials, which suggests a general tendency in the dataset.

### Dataset application in IE comparative studies

We demonstrated the intended deployment of PV600 by evaluating the performance of different approaches to IE. As explained in the Methods section, we reviewed the capabilities of rule-based and QA tools and compared them to free and paid generative language models on the same task. Prior to IE, we preselected snippets that contain relevant information with the aim to explore the benefits of including this step in IE procedures.

Snippet preselection can only be performed with generative language models, because QA tools were not trained for this task and are uncapable to preselect snippets. At the outset, we compared the behaviour of free and paid models on preselection independently of IE. Our target was to identify all the snippets where a bandgap value had been annotated. Prompt engineering was carried out iteratively, as described in SI section S4, and we discovered that distinct prompts were optimal for each of the generative models tested. The behaviour of Mixtral and Mixtral-Instruct models was significantly different, for example, which means that training the Mixtral-Instruct from Mixtral to receive instructions had affected how the model classifies the results. Pre-selection accuracy was evaluated on the gold standard truth to compute the metrics in the Table 4. By far the best performing model in preselection was GPT-4o with the F1-score of 91.6  ± 0.2. The F1-scores of other models ranged between 44.6 and 63.4. Llama-3.1 and Llama3-ChatQA demonstrated high recall, indicating that the models tend to determine most of the texts with a bandgap value; but since precision was low, many of the snippets without a correct value were also selected.

**Table 4:** Preselection evaluation results precision (P), recall (R) and F1-score (F1) of LLMs.

	Mixtral	Mixtral-Instruct	Llama-3.1	Llama3-ChatQA	GPT-4o
P	33.7	74.3	32.8	35.8	87.8 (±0.6)
R	66.0	55.3	92.0	97.3	94.7 (±1.8)
**F1**	**44.6**	**63.4**	**48.3**	**52.4**	**91.6** (±**0.2**)

The GPT-4o results are presented as average  ± standard deviation.

Next, we proceeded to investigate the differences between the three proposed IE protocols. In Test 1, we continued on from the preselection test outlined above and appended an IE task. For each of the language models, their preselected subset of snippets underwent IE with the same language model. This procedure illustrates the power of using individual generative language models throughout (QA and rule based tools were excluded). The trends in model performance, found in the first part of Table 5, were comparable to the preselection outcomes. GPT-4o achieved the highest F1-score (81.4). Second-best performance was observed with Llama3-ChatQA and Mixtral-Instruct with F1 near 53%, which was much better than base models of their type. These findings emphasise the sizable benefits of further training of open models towards questions and instructions in IE.

**Table 5:** IE results from the three tests for all the different models.

	Metric	Mixtral	Mixtral-Instruct	Llama-3.1	Llama3-ChatQA	GPT-4o	CDE2	QA-MatSciBERT
T1	P	23.4	67.1	23.8	44.0	81.7 (± 0.2)	—	—
	R	41.0	43.2	54.6	67.8	81.1 (± 0.4)	—	—
	F1	**29.8**	**52.6**	**33.2**	**53.4**	**81.4** (± **0.3**)	—	—
T2	P	71.2	79.9	77.8	77.7	81.7 (± 0.2)	87.0	87.5
	R	59.9	71.8	60.3	69.2	81.1 (± 0.4)	29.5	61.7
	F1	**65.1**	**75.6**	**68.0**	**73.2**	**81.4** (± **0.3**)	**44.1**	**72.3**
T3	P	23.0	32.8	25.6	41.0	65.6 (± 0.6)	81.6	65.0
	R	63.9	75.3	61.2	71.4	83.1 (± 1.3)	31.3	63.0
	F1	**33.8**	**45.7**	**36.0**	**52.1**	**73.3** (± **0.9**)	**45.2**	**64.0**

T1 stands for Test 1 (preselection with the same model), T2 for Test 2 (preselection with GPT-4o) and T3 for Test 3 (IE without preselection). The GPT-4o results are presented as average  ± standard deviation.

In Test 2, the same snippets preselected with GPT-4o served to initiate the IE task with all the models tested. As seen in Table 5, this approach generally improved the performance of open generative models due to GPT-4o’s effective preselection. IE with open models Llama3-ChatQA and Mixtral-Instruct now approached the paid GPT-4o with up to 75.6% in F1-score. While the recall of Llama-3.1 and Llama3-ChatQA remained relatively unchanged compared to Test 1, their precision improved substantially. This suggests that without preselection, these models tend to classify nearly all snippets as containing bandgap values, leading to lower precision. In other IE approaches, the rule-based CDE2 method demonstrated high precision (87.0) but lower recall, while QA-MatSciBERT achieved an F1-score of 72.4, comparable to Llama3-ChatQA.

Pre-selection was omitted in Test 3, allowing the models to process the entire pool of snippets and fully demonstrate their IE capabilities. Across the LLMs, F1-scores were generally lower than in Test 2. Remarkably, the GPT-4o F1-score was lower (73.3  ± 0.9) than with the preselection (81.4  ± 0.3), which indicates that the model has not in Test 3 first ensured that the values is present in the snippet and reflects the internal reasoning of the model. The CDE2 produced slightly higher F1-scores in Test 3, caused by an increase in recall despite a decline in precision. and a similar trend with precision and recall was observed with QA-MatSciBERT. However, with QA-MatSciBERT, the increase in precision did not compensate for the lower recall, resulting in a higher F1-score with Test 2 and preselection. Despite this, in Test 3 QA-MatSciBERT achieved the second-highest F1-score (64.0) behind GPT-4o.

**Fig. 7:**
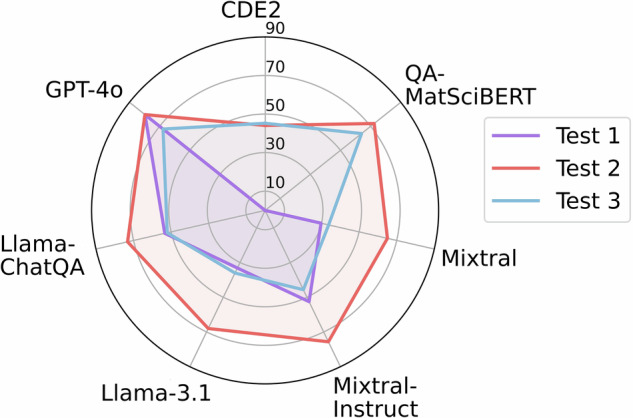
The F1-scores of the three IE tests illustrated for all the different models and methods used in the tests.

The F1-scores visualised in Fig. 7 suggest that best quality IE overall was achieved with the Test 2 procedure. This implies that the preselection produced benefits only if highly accurate, requiring the use of paid models. However, a close inspection reveals that in the case of QA-MatSciBERT and GPT-4o, there is very little difference in IE with and without preselection. For users constrained to free IE tools, QA-MatSciBERT would present an alternative to GPT-4o, with no need for additional preselection IE steps.

Lastly, we assessed the performance of the most powerful LLM (GPT-4o) in predicting bandgap type. The prompt was designed according to the following principles: the classification of bandgap types should be achievable solely using the rules provided in the prompt, and the predicted types should be of similar format for each snippet. Prompt optimisation is described in the SI section S6.

The accuracy of GPT-4o in predicting the correct bandgap type was 73.7 ± 1.4 % overall (an average from three repetitions). The results for all types were reported in SI Table S8, and reveal that the model performed best in classifying the computational values (97.1 ± 0.0 % correct). The bandgaps from the literature and from unknown source were classified with similar accuracy (71.6 ± 0.0 % and 72.0 ± 0.7 %), but classification of the experimental values fell short with only 62.2 ± 4.4 % accuracy. The underlying cause was misclassification of experimental bandgaps as computational ones, in the cases where the values were calculated from experimental plots (see SI for details).

As demonstrated in this study, the perovskite bandgap literature dataset serves as a versatile resource for evaluating IE methods. It can be utilised not only to assess the ability of these methods to extract bandgap values, but also to examine and compare model behaviour in detail. Furthermore, the dataset provides insight into historical trends in the studies of perovskites and highlights variations across different materials. By introducing the dataset, along with the steps needed to produce it, we provide a framework for developing further annotated datasets for the advancements of NLP approaches in the materials science community.

## Usage Notes

The data is organised in one folder and one csv table in a Zenodo repository^46^. The folder *PV600* includes ascii format *.txt snippets and the *brat* *.ann files with the annotations. These both filetypes can be accessed with text editors, but they are best processed with the *brat* program.

## Supplementary information

Supplementary Information

## Data Availability

All codes used to construct the annotated dataset and to perform the technical validation are publicly available^57^.
