# Supplementary material for: Annotated textual dataset PV600 of perovskite bandgaps for information extraction from literature
Source: Sci Data. 2025 Aug 11;12:1401. doi: 10.1038/s41597-025-05637-x (PMC12339702; doi:10.1038/s41597-025-05637-x)
Supplement: Supplementary file 1 — Supplementary Information [file 41597_2025_5637_MOESM1_ESM.pdf]

Supplementary Information
for
Annotated textual dataset PV600 of perovskite bandgaps for information extraction from literature

M. Sipilä[1], F. Mehryary[2], S. Pyysalo[2], F. Ginter[2] and M. Todorović[1]
[1] *Department of Mechanical and Materials Engineering, University of Turku, Finland*
[2] *Department of Computing, University of Turku, Finland*

S1. SELECTING THE SNIPPETS FOR ANNOTATION

From the corpus of full text manuscripts, we set out to identify open access manuscript for further processing into text snippets. All arXiv and Core publications were already open access, but from other three publishers (Elsevier, Springer and Royal Society of Chemistry, RSC) we identified the open access ones. Information about open access was marked by tags or text strings for Elsevier, Springer and RSC as follows:

- Elsevier: XML tag "<openaccessArticle>true</openaccessArticle>"
- Springer: XML tag "bodyhtml-grant</meta-name><meta-value>OpenAccess</meta-value>"
- RSC: Text string "This Open Access Article is licensed under".

Table S1 describes how many full texts from the total corpus were open access, classified by publisher. For our specific search on "perovskite" materials, the Springer corpus was almost 30% open access, in contrast to Elsevier where only 3% or the articles were open access.

TABLE S1: Number of total full texts downloaded and the proportion of open access texts by publisher.

Publisher	Number of publications	Number of open access publications	Percentage of open access publications
Elsevier	113,438	3,484	3.1 %
Springer	34,379	11,330	33.0 %
RSC	16,634	3,003	18.1 %
Core	27,500	27,500	100 %
arXiV	2,371	2,371	100 %
Total	194,322	47,688	24.5 %

Starting with the open access corpus, we proceeded to select the 600 snippet dataset. The focus was on three hybrid perovskites (MAPI, FAPI and MAPB) and two inorganic halide perovskites ($CsPbI_3$ and $CsPbBr_3$). An equal number of snippets per material (120 each) was distributed across the publishers, as much as the content allowed, as described in Table S2.

TABLE S2: Number of material-specific snippets selected from each publisher so that each column totals 120.

Publisher	MAPI	FAPI	MAPB	$CsPbI_3$	$CsPbBr_3$
Elsevier	24	19	19	27	24
Springer	24	24	19	14	24
RSC	24	26	27	26	24
Core	24	26	28	27	24
arXiV	24	25	27	26	24

S2. ANNOTATION GUIDELINES AND PROCEDURES

To evaluate the performance of IE methods on extracting bandgaps, it is first necessary to determine the ground truth for each snippet through human annotation. While the annotators are materials science experts, there is still a significant risk of individual misinterpretation or differing interpretations of the snippets. To ensure high-quality annotations, the process must be technically straightforward, supported by clear instructions, and accompanied by case examples. The purpose of the annotation guideline document is to establish the rules of annotation and ensure that all of the experts are performing this task in a similar way. Below, we describe the considerations related to the annotation implementation and instructions. The Annotation Guideline full text is publicly available in the PV600 repository.

To begin with, the annotators had to become familiar with the annotation tools and file organisation for their task. We followed the recommended procedure with the *brat* software [1]. This tool is easy to use, does not require annotators to install any software and is easily modifiable for different annotation tasks. The *brat*-program was downloaded [2] and installed on an external server. The content (illustrated in Figure S1 a.) included the snippets in the Bandgap600 folder, the *brat* native examples and custom-made tutorials for annotators. Each annotator had access to this content from their web browser, and a username and password were provided to each user to protect the snippets and annotations.

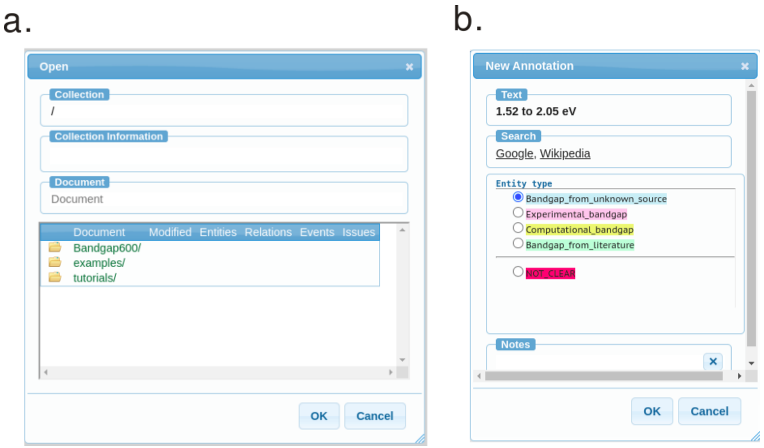

FIG. S1: The *brat* windows. a. Example of the *brat* folders in an user view in the browser window. b. The *brat* annotating dialogue.

The snippet files were organised into folders for each annotator, and then divided by the material of interest. The objective of the annotation was to find the correct answer to the annotation question and as a rule, the bandgap to be annotated should be the bandgap of the material labeled in the snippet. The following general rules were also applied:

- Annotated entity should contain numbers.
- The unit of the annotated value should be in electronvolts.
- There can be more than one bandgap value in the one snippet.
- The type of the bandgap should be determined by the origin of the bandgap, if it is explained in the snippet.

To perform the annotation, the annotators selected the text span by mouse to create an annotation instance. Once the user had generated the instance, also called an entity, the type of this entity could be selected. As presented in Figure S1 b, we configured *brat* to allow annotation of four different bandgap types. In the case the type was difficult to decide or the annotator was unsure about the text, they were advised to select the NOT CLEAR option.

After stating the general rules, we provided the annotators with multiple examples of different correct and incorrect cases they might encounter during the annotation procedure. The examples varied from cases, where there was a range (e.g. 1.5-1.6 eV), to error margin cases (e.g. 2.3 $\pm$ 0.1 eV) and to cases where there was multiple bandgap values in the one snippet (e.g. 1.5 eV, 1.6 eV and 1.55 eV in the same snippet). We also provided examples of negative cases, which should be left unannotated: for example the case when the bandgap value does not belong to the material of the snippet. The examples contained also the information about the bandgap type and which type to select in different contexts.

S3. DATASET APPLICATION TO IE TASKS: INTRODUCTION TO IE TOOLS

The CDE2 is a multipurpose package designed to extract information from materials science, physics and chemistry articles. Its information extraction approach is based on predefined sentence structures with additional probabilistic Snowball algorithm. It has been used to extract information from photocatalytic water splitting procedures [3], semiconductor bandgaps [4], Curie and Néel temperatures [5] and battery materials [6], among others. We followed the procedure and postprocessing steps recommended in previous work [4]. The CDE2 by default extracts all of the bandgaps of all of the materials, so we selected only those answers returned which corresponded to the material in the snippet, to reliably compare the different methods.

The QA-MatSciBERT method is based on the MatSciBERT language model fine-tuned for the question answering task with SQuAD2 dataset [7]. The user asks a question from a context document and the model, a generic factual answer extractor, selects the most probable text span from the document to answer the question. Previous work has reviewed QA performance based on different BERT models [8] and MatSciBERT [9] was discovered to perform best, so we included it in this comparison.

The generative language models are not explicitly targeted towards IE tasks, but as multi-purpose models they are capable to extract information from provided snippets by using them as prompt inputs and generating the answer. For comparative studies, we selected four open source and free generative language models (Mixtral, Mixtral-Instruct, Llama-3.1 and Llama3-ChatQA) and one paid language model (GPT-4o).

The Mixtral is a generative sparse mixture-of-experts language model with open weights. It outperforms Llama 2 70B [10] and GPT-3.5 [11] performance on most benchmarks [12]. The Mixtral-Instruct was trained from base Mixtral using supervised fine-tuning to follow instructions. Llama-3.1 is an auto-regressive language model that uses an optimised transformer architecture. It is competitive with the leading generative models, including GPT-4 and GPT-4o [13]. The Llama3-ChatQA model was built on top of the Llama3-model to excel at conversational question answering and retrieval-augmented generation [14]. The GPT-4o model is a large multimodal generative model for complex, multi-step tasks [15]. All the models were executed using fixed seed and zero temperature to ensure deterministic output. Even with these, the GPT-4o model exhibited non-deterministic behaviour, which was also documented by developers [16, 17]. We repeated all the computations with the GPT-4o three times and report the average and the standard deviation of the results. Other models did not exhibit such variation, so we only executed them once.

Because generative language models tend to return more than just the numerical span from the snippet, post-processing was employed to remove extra text around the returned answers, which is standard practice in the IE applications. Generative models, unlike QA-MatSciBERT or CDE2, also have an inherent property to hallucinate answers if the correct answer can not be found from the snippets. The QA-MatSciBERT and CDE2 are, by default, incapable of generating text which does not appear in the input documents. The hallucinated values were not removed to facilitate a factual comparison.

S4. DATASET APPLICATION TO IE TASKS: SNIPPET PRESELECTION

To improve the accuracy of IE workflows, we tested the capability of generative language models to identify the snippets that contain a bandgap value over those that don't. Because each of the generative language models had been trained differently, we carried out a prompt optimisation study to discover the best prompts for each model, as compared to the annotated ground truth. We constructed the prompts from three parts: Question, Instructions and the Context, as follows:

1. Question: the role the first prompt was to indicate interest in "numerical values of bandgap(s)" for specific materials.

2. Instruction: the role of the second prompt was to affect the manner of reporting the answers. For example, for some generative models it made a difference whether one of the instructions was 'Answer *only* Yes or No' or 'Answer *always* Yes or No', because the first format could lead also to empty answers.

3. Context: this prompt segment served to input the context document (snippet) that the operation was to be performed on. For Mixtral, Mixtral-Instruct and GPT-4o this was formulated as *"Context: "+text+" Answer:"*.,

TABLE S3: Prompts for preselections.

	Question	Instructions
P1	Does the following context contain the numerical bandgap value or bandgap values of "+material+"?	Answer 'Yes' or 'No'.
P2	Does the following context contain the numerical bandgap value or bandgap values of *pure* "+material+"?	Answer only Yes or No.
P3	Does the following context contain the numerical bandgap value or bandgap values of *pure* "+material+"?	Answer is 'Yes' or 'No'. Answer always 'Yes' or 'No'.
P4	Does the following context contain the numerical bandgap value or bandgap values of *pure* "+material+"?	Answer 'Yes' or 'No'. Answer always 'Yes' or 'No'.
P5	Does the following context contain the numerical bandgap value or bandgap values of *pure* "+material+"?	Answer is Yes or No. Answer Yes only if you are sure that the bandgap value belongs to pure "+material+".
P6	Does the following context contain the numerical value of bandgap or *numerical values* of bandgaps of *pure* "+material+"?	Answer only 'Yes' or 'No'.
P7	Does the following context contain the numerical value of bandgap or *numerical values* of bandgaps of *pure* "+material+"?	Answer always 'Yes' or 'No'.
P8	Does the following context contain the numerical value of bandgap or *numerical values* of bandgaps of *pure* "+material+"?	Answer only 'Yes' or 'No.' Answer always 'Yes' or 'No'.

where *"+text+"* denoted the snippet text. The last item (*"Answer:"*) in the context indicates to the generative model that the generated text should be the answer. For Llama3.1 and Llama-ChatQA we followed the input formatting scheme as suggested in the model description [18], because in the previous tests this was found to perform the best with these models.

Table S3 features a selection of 8 combinations of Questions and Instructions tested to maximise the accuracy of snippet preselection. We restricted the trials to 8 prompt iterations after we observed sufficient variation in the responses of language models. The Question in prompt 1 is clear and concise. After it we added the word *pure* (bandgap should belong to the pure material, not mixture) to the Question and kept the Question in prompts 2-5 same. In the Question used in prompts 6-8 we additionally requested that 'numerical values' be returned for bandgaps in plural.

For the Instructions, the changes were small but different combinations were tested because even slight alterations in wordings changed the output. For example if the instruction contained the word 'only' ('Answer only Yes or No'), the models were more likely to return only the word 'Yes' or 'No'; if this was not defined, the models had a tendency to add more text to the answer.

Test outcomes for 5 generative models are presented in Table S4, with the best F1-score for each model indicated in bold. We observed notable variation in selection accuracy with different prompts. The range of variations between the F1-scores was small for GPT-4o model, which also exhibited the best performance overall with an F1-score of 91.60 (± 0.23). This model was very confident in the answers, so minor changes in reporting them did not make a large difference. The next best F1-score of 63.41 was obtained with the Mixtral-Instruct model, where different prompts led to 50% - 70% differences in outcomes. This volatility between the prompts is clear also for the base Mixtral model, where the model had F1-score of 44.60 with the prompt 3 and only 13.71 with the prompt 6. In this case small differences in the instructions greatly affected the performance of the model: with the prompt 3 the model did not always return something, but with the prompt 2 (Answer always 'Yes' or 'No') the model was forced to always return a value. This emphasises that even small deviations between prompts affects the end result. The Llama-3.1 and Llama-ChatQA models were more consistent in their results, but it is notable that these models constantly have high recall. This indicates that the Llama models have had a tendency to classify more snippets as containing bandgap values than actually true. This may originate from the model training, or the models may consider any numerical value present in the snippet to be the bandgap value.

Another finding is that the models performed best with different prompts, which underlines the importance of prompt optimisation and model-specific prompts. Only prompt 5 was identified as optimal for more than one model (Mixtral-Instruct and Llama-ChatQA). This prompt contained an instruction that the model should answer 'Yes' only if sure about it, which may indicate that some models require endorsing the certainty of the model.

TABLE S4: Pre-selection accuracy with 8 different prompts for 5 generative models tested. The evaluation metrics are F1-score (F1), precision (P) and recall (R).

	Mixtral	Mixtral-Instruct	Llama-3.1	Llama-ChatQA	GPT-4o
P1 P	26.84	74.29	31.26	31.65	79.67 (± 6.68)
R	32.98	27.66	96.28	93.62	96.81 (± 4.15)
F1	29.59	40.31	47.20	47.31	87.18 (± 2.13)
P2 P	27.41	75.71	32.77	33.33	87.74 (± 1.14)
R	37.77	28.19	92.02	96.28	94.86 (± 3.38)
F1	31.77	41.09	**48.32**	49.52	91.13 (± 0.97)
P3 P	33.70	69.23	31.64	33.46	87.79 (± 1.52)
R	65.96	23.94	99.47	95.21	92.73 (± 1.54)
F1	**44.60**	35.57	48.01	49.52	90.17 (± 0.13)
P4 P	28.93	76.74	31.53	35.01	88.09 (± 2.19)
R	48.94	17.55	96.28	92.55	93.97 (± 1.23)
F1	36.36	28.57	47.51	50.80	90.92 (± 0.57)
P5 P	30.44	74.29	33.13	35.81	86.92 (± 3.12)
R	57.98	55.32	85.64	97.34	95.39 (± 3.38)
F1	39.92	**63.41**	47.77	**52.36**	90.88 (± 0.11)
P6 P	28.33	63.43	32.61	34.30	87.09 (± 2.37)
R	9.04	45.21	63.83	81.91	93.62 (± 4.15)
F1	13.71	52.80	43.17	48.35	90.16 (± 0.83)
P7 P	21.66	59.87	31.57	31.72	87.81 (± 0.61)
R	25.00	50.00	98.40	97.87	94.68 (± 1.84)
F1	23.21	54.49	47.80	47.92	**91.60 (± 0.23)**
P8 P	33.85	63.16	31.68	32.32	84.39 (± 5.98)
R	11.70	51.06	91.49	96.28	94.50 (± 0.81)
F1	17.39	56.47	47.06	48.40	89.07 (± 3.17)

S5. DATASET APPLICATION TO IE TASKS: EXTRACTING BANDGAP VALUES

In the comparative tests of bandgap extraction capabilities, suitable prompts were needed for the 5 generative language models tested, and in some cases, for the QA approach as well. We carried out limited prompt testing to optimise the IE performance of these models, before the final comparison was made. We designed four different prompts, ranging from general (prompt P1) to detailed (prompt P4), to explore model responses to varying level of detail in the prompt. The text [material] was substituted with the material of interest. With the models Mixtral, Mixtral-Instruct, GPT-4o and QA models after the prompts, the word *"Context"* was followed by the snippet. For Llama3.1 and Llama-ChatQA we followed the model input formatting scheme [18], where the snippet was used as context. When using the generative models Mixtral, Mixtral-Instruct and GPT-4o, after the snippet text we also provided last text *"Answer:"*, which guides the generative model to generate an answer next.

- P1: "What is the numerical bandgap value of [material] in the following context?"
- P2: "What is the numerical value of bandgap, or values of bandgaps, of pure [material] in the following context?"
- P3: "In the following context, what is the numerical bandgap value or bandgap values of pure [material]? Only answer a single number in eV units or, in case of multiple reported bandgaps, numbers in eV units. Answer 'none' if there is no statement about the bandgap, or if there is no statement of numerical bandgap."
- P4: "In the following context, what is the absolute bandgap value or bandgap values of pure [material]? Only answer a single number in eV units or, in case of multiple reported bandgaps, numbers in eV units. Answer 'none' if there is no statement about the bandgap, or if there is no statement of absolute bandgap. Do not report bandgaps of doped materials, or if the text does not indicate to which material the bandgap belongs."

Test 1 featured only generative language models, and the same model was applied to both snippet preselection and the IE task. Preselection prompts were set as described in the section S4, and the IE task prompts were selected based on the prompt optimisation test outcomes presented in Table S5. Again the GPT-4o performed best with at F1-score of 81.36 (± 0.28). The GPT-4o F1-scores ranged from 62.67 (± 8.53) (prompt 2) to 81.36 (± 0.28) and 79.57 (± 0.84) (prompts 3 and 4) with more detailed prompts, which indicates that the model excels with longer prompts. The second best results were achieved with Llama-ChatQA and Mixtral-Instruct, with very similar F1-scores of 53.38 and

TABLE S5: Test 1 evaluation results with prompts 1-4 for all generative models. Best prompt results are indicated in bold for each model.

	Mixtral	Mixtral-Instruct	Llama-3.1	Llama-ChatQA	GPT-4o
P1 P	23.37	65.96	23.80	39.80	73.54 (± 5.56)
R	40.97	40.97	54.63	69.60	63.29 (± 0.67)
F1	**29.76**	50.54	**33.16**	50.64	66.72 (± 0.59)
P2 P	19.59	67.12	23.09	44.00	69.25 (± 11.49)
R	37.89	43.17	51.98	67.84	59.47 (± 8.78)
F1	25.83	**52.55**	31.98	**53.38**	62.67 (± 8.53)
P3 P	17.18	62.22	22.41	35.31	81.66 (± 0.22)
R	39.65	24.67	53.30	68.28	81.06 (± 0.44)
F1	23.97	35.33	31.55	46.55	**81.36 (± 0.28)**
P4 P	17.68	64.29	22.18	33.49	82.39 (± 0.40)
R	37.00	23.79	52.86	64.76	76.94 (± 1.27)
F1	23.93	34.73	31.25	44.14	79.57 (± 0.84)

52.55 obtained with prompt 2. The poorest preselection was obtained with the Mixtral and Llama-3.1 models. They performed best with the shortest prompt (prompt 1) suggesting an inability to process multiple sentence prompts.

In Test 2, snippets preselected with GPT-4o served to initiate IE with all the models. From the three executions of preselection results with GPT-4o we selected the one which yielded to F1-score closest to the reported average. Prompt engineering was also necessary for the QA model. Our observations are summarised in Table S6. The F1-scores for all of the models improved dramatically from Test 1, which is explained by the better preselection quality. The best improvement was found with the Mixtral model, where the F1-score increased from 29.76 to 65.07 - this underlines the difference in preselection quality with Mixtral and GPT-4o. Given that most snippets now had values, the simple prompt 1 was the best IE prompt for Mixtral, Llama-ChatQA and QA based on MatSciBERT. The second most simple prompt 2 performed best with Llama-3.1 and Mixtral-Instruct. Mixtral-Instruct with prompt 2 achieved a score of 75.64, approaching GPT-4o performance of 81.36 (± 0.28).

TABLE S6: Test 2 evaluation results with prompts 1-4 for all generative models and QA. Best prompt results are indicated in bold for each model. The GPT-4o results are the same as in the Test 1.

	Mixtral	Mixtral-Instruct	Llama-3.1	Llama-ChatQA	GPT-4o	QA
P1 P	71.20	81.35	66.96	77.72	73.54 (± 5.56)	87.50
R	59.91	69.16	66.08	69.16	63.29 (± 0.67)	61.67
F1	**65.07**	74.76	66.52	**73.19**	66.72 (± 0.59)	**72.35**
P2 P	56.03	79.90	77.84	77.00	69.26 (± 11.49)	85.21
R	57.27	71.81	60.35	67.84	59.47 (± 8.78)	53.30
F1	56.64	**75.64**	**67.99**	72.13	62.67 (± 8.53)	65.58
P3 P	62.14	78.29	63.68	80.23	81.66 (± 0.22)	83.33
R	56.39	44.49	53.30	62.56	81.06 (± 0.44)	8.81
F1	59.12	56.74	58.03	70.30	**81.36 (± 0.28)**	15.94
P4 P	59.30	75.40	62.87	81.32	82.39 (± 0.40)	85.71
R	51.98	41.85	46.26	65.20	76.94 (± 1.27)	5.29
F1	55.40	53.82	53.30	72.37	79.57 (± 0.84)	9.96

In test 3 there was no preselection, this was the standard IE benchmark scenario. The results were again optimised over four prompts and showcased in the Table S7. The F1-scores of all models were lower than in Test 2, highlighting the beneficial impact of preselection on information extraction (IE) tasks. Even GPT-4o exhibited a drop in model performance. The highest F1-score was still achieved by GPT-4o (73.31 (± 0.86)), while the QA model performed comparably with an F1-score of 63.98. The Mixtral and Llama-3.1 models yielded better results without preselection (with the same model), likely due to limited capability for classifying snippets. Although Test 3 was tougher than previous tasks, the two simplest ones (prompt 1 and 2) again performed best for almost all models, including QA. A notable exception was GPT-4o, where complex prompts 3 and 4 still produce the best performance. These observations suggest that optimal prompts for each model depend more on the details of their training than then complexity of the IE task.

TABLE S7: Test 3 results with prompts 1-4 (no preselection). Best prompt results are indicated in bold for each model.

	Mixtral	Mixtral-Instruct	Llama-3.1	Llama-ChatQA	GPT-4o	QA
P1 P	22.98	32.76	22.48	37.33	36.52 (± 0.07)	65.00
R	63.88	75.33	68.72	72.69	67.25 (± 0.67)	63.00
F1	**33.80**	**45.66**	33.88	49.33	47.34 (± 0.20)	**63.98**
P2 P	18.38	31.60	25.55	41.01	36.63 (± 0.23)	62.56
R	60.79	77.53	61.23	71.37	67.84 (± 1.16)	53.74
F1	28.22	44.90	**36.06**	**52.09**	47.58 (± 0.42)	57.82
P3 P	15.38	29.81	21.63	30.78	61.81 (± 0.06)	68.97
R	59.91	48.46	48.02	69.16	87.22 (± 0.45)	8.81
F1	24.48	36.91	29.82	42.61	72.35 (± 0.17)	15.62
P4 P	15.75	31.85	22.41	30.02	65.58 (± 0.64)	85.71
R	55.51	44.05	47.58	57.27	83.11 (± 1.27)	5.29
F1	24.54	36.97	30.47	39.39	**73.31 (± 0.86)**	9.96

S6. DATASET APPLICATION TO IE TASKS: EXTRACTING BANDGAP TYPES

In the final test, best performing generative model GPT-4o was required to classify bandgap type between experimental, computational, literature and unknown categories. Here, a very detailed and structured prompt was necessary: instead of identifying bandgaps, the models were presented with pre-identified bandgap values, then tasked with selecting the type. We designed two approaches for introducing bandgap values: prompt 1 used the initial and final characters of the annotation, and prompt 2 relied on the ordinal number of the bandgap annotated in the snippet. By this we tried to assess the problem of the GLMs tendency to underperform in numerical tasks. The final prompt had five components, indicated below:

Prompt 1

(a) ”What is the type of the bandgap ’value’ (beginning index ’beginning index’ and ending index ’ending index’ in the context) in the following context?”

(b) ”Context:” + snippet +

(c) ”The bandgap type is either ’Experimental’, ’Computational’, ’Literature’ or ’Unknown’.”

(d) ”Answer as a list of lists with the bandgap type and the beginning index. For example: [[’Experimental’, 235], [’Literature’], 345], where the ’Experimental’ is the bandgap type for the bandgap with beginning index 235 and ’Literature’ is the bandgap type for the bandgap with beginning index 345.”

(e) : ”The ’Experimental’ is bandgap which has been measured in experimental means in the context document. The ’Computational’ is bandgap which has been obtained in theoretical or computational means in the context document. The ’Literature’ type is the bandgap value which originates from some other article (this can be determined by reference or reference brackets). The ’Unknown’ bandgaps are bandgaps from which it is not possible to deduce how they were obtained.”

Here, (a) describes the main question, but augmented with the bandgap location in text (denoted by initial and final character index). This was to account for multiple bandgap values potentially present in the context document, which was indicated in segment (b). Classification categories were introduced in (c) and explained to the model in (e). Lastly (d) served to instruct the model about the desired answer format. The final Prompt1 formulation was:

Prompt 1 = ”What is the type of the bandgap ’value’ (beginning index ’beginning index’ and ending index ’ending index’ in the context) in the following context? Context: ” + snippet + ”The bandgap type is either ’Experimental’,

'Computational', 'Literature' or 'Unknown'. Answer as a list of lists with the bandgap type and the beginning index. For example: [['Experimental', 235], ['Literature'], 345], where the 'Experimental' is the bandgap type for the bandgap with beginning index 235 and 'Literature' is the bandgap type for the bandgap with beginning index 345. The 'Experimental' is bandgap which has been measured in experimental means in the context document. The 'Computational' is bandgap which has been obtained in theoretical or computational means in the context document. The 'Literature' type is the bandgap value which originates from some other article (this can be determined by reference or reference brackets). The 'Unknown' bandgaps are bandgaps from which it is not possible to deduce how they were obtained."

In Prompt 2, only statements (a) and (d) were altered to remove references to initial and final index. Instead, the ordinal number of appearance in the list of bandgap annotations was used to select the bandgap to be classified, as illustrated below.

(a) What is the type of the bandgap 'bandgap value' ('ordinal value' bandgap value of 'material' in the context) in the following context?

(b) *same as Prompt 1*

(c) *same as Prompt 1*

(d) Answer as a list of lists with the bandgap type and the ordinal number of the bandgap value. For example: [['Experimental', 1], ['Literature'], 2], where the 'Experimental' is the bandgap type for the first bandgap value in the snippet and 'Literature' is the bandgap type for the second bandgap value in the snippet.

(e) *same as Prompt 1*2

Prompt 2 = What is the type of the bandgap 'bandgap value' ('ordinal value' bandgap value of 'material' in the context) in the following context? Context: + snippet +. The bandgap type is either 'Experimental', 'Computational', 'Literature' or 'Unknown'. Answer as a list of lists with the bandgap type and the ordinal number of the bandgap value. For example: [['Experimental', 1], ['Literature'], 2], where the 'Experimental' is the bandgap type for the first bandgap value in the snippet and 'Literature' is the bandgap type for the second bandgap value in the snippet. The 'Experimental' is bandgap which has been measured in experimental means in the context document. The 'Computational' is bandgap which has been obtained in theoretical or computational means in the context document. The 'Literature' type is the bandgap value which originates from some other article (this can be determined by reference or reference brackets). The 'Unknown' bandgaps are bandgaps from which it is not possible to deduce how they were obtained.

Test outcomes in Table S8 illustrate the proportion of correct classifications (diagonal, in bold) and various kinds of misclassifications (in columns, per category) for Prompts 1 and 2. The columns indicates the ground truth total bandgap counts in each category. Overall, we observed as averaged from three executions that classification accuracy of predicted bandgap types was 73.0 (± 1.5) % with the Prompt 1 and 73.7 (± 1.4) % with the Prompt 2 (calculated as the proportion of correctly predicted values across all categories and the total count of bandgaps). The better performance with Prompt 2 suggests that GPT-4o can process ordinal numbers better than nominal ones, which is expected given that the model has been trained more on the texts than mathematical operations.

The model performed very well when classifying computational bandgaps, with an accuracy of 97.1 (± 0.0) %. The classification accuracy for other bandgap types ranged between 62.2 (± 4.4) % and 72.0 (± 0.7) %, with experimental bandgaps being the least accurately classified. A closer look at misclassified values revealed that most often the experimental value was mistaken for computational (7.7 (± 0.6) out of 45 cases). We found there was a clear reason for this prediction: in the 8 cases the bandgap had been calculated from the experimental results, for example using Tauc's plot to compute the bandgap value from the measured absorption spectrum of the material. Other notable prediction errors were related to difficulties in distinguishing between bandgaps from literature and unknown sources (average 25.7 cases of erroneous classification). However, this needn't be an issue in practice: in many applications it would be important to identify if the bandgaps were experimental or computational, and other bandgap types could be merged into a single category.

TABLE S8: Classification accuracy of bandgap types with GPT-4o. The number of predicted values is followed in parentheses by the corresponding fraction [%] from the total count in each category (ground truth). Values in bold represent correctly classified bandgap types, with all other values describing misclassification.

		EG_{EXP}	EG_{COMP}	EG_{LIT}	EG_{UNK}
P1	EG_{EXP}	**23.7 ± 0.6 (53.7 ± 1.3)**	0.0 ± 0.0 (0.0 ± 0.0)	3.7 ± 0.6 (5.5 ± 0.9)	2.7 ± 0.6 (3.3 ± 0.7)
	EG_{COMP}	9.7 ± 0.6 (21.6 ± 1.3)	**32.0 ± 0.0 (94.1 ± 0.0)**	0.7 ± 1.2 (1.0 ± 1.8)	3.7 ± 0.6 (4.6 ± 0.7)
	EG_{LIT}	3.7 ± 1.2 (8.2 ± 2.7)	1.3 ± 0.6 (3.8 ± 1.8)	**52.7 ± 3.2 (78.7 ± 4.8)**	17.3 ± 1.5 (21.4 ± 1.9)
	EG_{UNK}	7.3 ± 1.2 (16.2 ± 2.7)	0.7 ± 0.6 (2.0 ± 1.8)	10.0 ± 1.0 (14.9 ± 1.5)	**57.3 ± 1.5 (70.7 ± 1.9)**
	Total	45 (100)	34 (100)	67 (100)	81 (100)
P2	EG_{EXP}	**28.0 ± 2.0 (62.2 ± 4.4)**	0.0 ± 0.0 (0.0 ± 0.0)	5.0 ± 0.0 (7.5 ± 0.0)	2.3 ± 0.6 (2.8 ± 0.7)
	EG_{COMP}	7.7 ± 0.6 (17.1 ± 1.3)	**33.0 ± 0.0 (97.1 ± 0.0)**	5.0 ± 0.0 (7.5 ± 0.0)	3.7 ± 0.6 (4.6 ± 0.7)
	EG_{LIT}	1.7 ± 0.6 (3.8 ± 1.3)	1.0 ± 0.0 (2.9 ± 0.0)	**48.0 ± 0.0 (71.6 ± 0.0)**	16.7 ± 1.5 (20.6 ± 1.9)
	EG_{UNK}	7.7 ± 1.5 (17.8 ± 3.3)	0.0 ± 0.0 (0.0 ± 0.0)	9.0 ± 1.0 (13.4 ± 1.5)	**58.3 ± 0.6 (72.0 ± 0.7)**
	Total	45 (100)	34 (100)	67 (100)	81 (100)

[1] P. Stenetorp, S. Pyysalo, G. Topić, T. Ohta, S. Ananiadou, and J. Tsujii, BRAT: A web-based tool for NLP-assisted text annotation, in *Proceedings of the Demonstrations at the 13th Conference of the European Chapter of the Association for Computational Linguistics* (2012) pp. 102–107.

[2] brat rapid annotation tool, `https://brat.nlplab.org`, accessed 2025-01-13.

[3] T. Isazawa and J. M. Cole, Automated construction of a photocatalysis dataset for water-splitting applications, Scientific Data **10**, 651 (2023).

[4] Q. Dong and J. M. Cole, Auto-generated database of semiconductor band gaps using ChemDataExtractor, Scientific Data **9**, 193 (2022).

[5] C. J. Court and J. M. Cole, Auto-generated materials database of Curie and Néel temperatures via semi-supervised relationship extraction, Scientific data **5**, 1 (2018).

[6] S. Huang and J. M. Cole, A database of battery materials auto-generated using ChemDataExtractor, Scientific Data **7**, 260 (2020).

[7] P. Rajpurkar, R. Jia, and P. Liang, Know what you don't know: Unanswerable questions for SQuAD, arXiv arxiv.org/abs/1806.03822 (2018).

[8] M. Sipilä, F. Mehryary, S. Pyysalo, F. Ginter, and M. Todorović, Question Answering models for information extraction from perovskite materials science literature. Preprint at https://arxiv.org/abs/2405.15290, (2024).

[9] P. Shetty, A. C. Rajan, C. Kuenneth, S. Gupta, L. P. Panchumarti, L. Holm, C. Zhang, and R. Ramprasad, A general-purpose material property data extraction pipeline from large polymer corpora using natural language processing, npj Computational Materials **9**, 52 (2023).

[10] H. Touvron, L. Martin, K. Stone, P. Albert, A. Almahairi, Y. Babaei, N. Bashlykov, S. Batra, P. Bhargava, S. Bhosale, *et al.*, Llama 2: Open foundation and fine-tuned chat models, arXiv preprint arXiv:2307.09288 (2023).

[11] GPT-3.5 Turbo, `platform.openai.com/docs/models/gpt-3-5-turbo` (), accessed 2024-06-05.

[12] Mixtral-8x7B, `mistral.ai/news/mixtral-of-experts/`, accessed 2024-10-21.

[13] Introducing Llama 3.1: Our most capable models to date, `ai.meta.com/blog/meta-llama-3-1/` (2024).

[14] Z. Liu, W. Ping, R. Roy, P. Xu, C. Lee, M. Shoeybi, and B. Catanzaro, ChatQA: Surpassing GPT-4 on conversational QA and RAG, Advances in Neural Information Processing Systems **37**, 15416 (2024).

[15] GPT-4o, OpenAI, `platform.openai.com/docs/models` (2024).

[16] OpenAI Reproducible outputs, `platform.openai.com/docs/advanced-usage/reproducible-outputs` (), accessed 2025-05-28.

[17] OpenAI Create chat completion, `platform.openai.com/docs/api-reference/chat/create` (), accessed 2025-05-28.

[18] Nvidia/Llama3-ChatQA-1.5-70B, `huggingface.co/nvidia/Llama3-ChatQA-1.5-70B`, accessed 2025-04-11.
